# Lagging Brain Gene Expression Patterns of *Drosophila melanogaster* Young Adult Males Confound Comparisons Between Sexes

**DOI:** 10.1007/s12035-024-04427-7

**Published:** 2024-08-28

**Authors:** Flannery McLamb, Zuying Feng, Jeanne P. Vu, Lindsey Griffin, Miguel F. Vasquez, Goran Bozinovic

**Affiliations:** 1 Boz Life Science Research and Teaching Institute, La Jolla, CA USA; 2 Division of Extended Studies, University of California San Diego, La Jolla, CA USA; 3 Graduate School of Public Health, San Diego State University, San Diego, CA USA; 4 National Center for Microscopy and Imaging Research, University of California San Diego, La Jolla, CA USA; 5 Center for Life in Extreme Environments, Portland State University, Portland, OR USA; 6 School of Biological Sciences, University of California San Diego, La Jolla, CA USA

**Keywords:** Brain, Transcriptome, Sexual dimorphism, Genomics

## Abstract

Many species, including fruit flies (*Drosophila melanogaster*), are sexually dimorphic. Phenotypic variation in morphology, physiology, and behavior can affect development, reproduction, health, and aging. Therefore, designating sex as a variable and sex-blocking should be considered when designing experiments. The brain regulates phenotypes throughout the lifespan by balancing survival and reproduction, and sex-specific development at each life stage is likely. Changes in morphology and physiology are governed by differential gene expression, a quantifiable molecular marker for age- and sex-specific variations. We assessed the fruit fly brain transcriptome at three adult ages for gene expression signatures of sex, age, and sex-by-age: 6698 genes were differentially expressed between sexes, with the most divergence at 3 days. Between ages, 31.1% of 6084 differentially expressed genes (1890 genes) share similar expression patterns from 3 to 7 days in females, and from 7 to 14 days in males. Most of these genes (90.5%, 1712) were upregulated and enriched for chemical stimulus detection and/or cilium regulation. Our data highlight an important delay in male brain gene regulation compared to females. Because significant delays in expression could confound comparisons between sexes, studies of sexual dimorphism at phenotypically comparable life stages rather than chronological age should be more biologically relevant.

**Supplementary Information:**

The online version contains supplementary material available at 10.1007/s12035-024-04427-7.

## Introduction

Differences between females and males in morphology, physiology, and behavior can have critical effects on reproduction and development [1–3], stress response [4, 5], health [6, 7], and aging [8, 9]. Many species exhibit observable and quantifiable sex-specific phenotypes [10–12], including larger body and cell size in female fruit flies, *Drosophila melanogaster* [13]. Sex is genetically determined within each fruit fly somatic cell based on X chromosome dosage: XX cells, which express the *Sex-lethal* (*Sxl*) gene, are female, and XY cells are male [14, 15].

Brain dimorphism contributing to sex-specific phenotypes is well-documented [16–18]. Adult male rats’ 18% larger ventral medial PFC is attributable to 13% fewer neurons and 18% fewer glia cells in females [19], and the male primary visual cortex has about 20% more gray matter volume, partially due to having 19% more neurons than females [20, 21]. Human male brains generally have larger volume, surface area, and white matter fractional anisotropy, while human female brains have greater raw cortical thickness, white matter tract complexity [22], and higher cerebral glucose metabolic rates [23]. Between ages 7 and 11, female subcortical forebrain nuclei reach adult volume, while males’ volume is greater but likely reduces later in adulthood [24]. Nerve fiber tract streamline reduction occurs earlier in females [25], while occipital area thinning is faster in males [26].

Many species are characterized by different maturation rates between sexes [27–31]. Direct temporal comparison of females and males is challenged by sex-specific phenotypic timelines, evident in quantifiable gene expression patterns. If sexes are compared at the same chronological rather than biological age, developmental changes may be misinterpreted as sexual dimorphism. Quantifying brain gene expression across life stages can identify developmentally comparable time points between females and males and characterize sex-specific physiology and behavior more comprehensively. Reproductive neurons in fruit flies manifest sex-dependent phenotypes. For example, the anterior dorsal neuronal (aDN) clusters [32] are responsible for collective egg laying and receiving olfactory inputs in females, whereas male aDN cells accept visual inputs and shape visual courtship behaviors. All *doublesex* + *(dsx* +*)* neuronal clusters are sexually dimorphic or sex-specific, as single-cluster mapping showed the absence of monomorphic clusters [32]. Nuances in sex- and age-specific effects in the brain can be quantified by analyzing phenotypes at the molecular level. Notably, some genes that affect sex-specific behaviors are not expressed within the brain [33]: the fat body around the brain likely modulates behavior [34] and contains sex-biased transcripts influencing sex determination pathways and brain gene expression [35]. While subtle anatomical dimorphisms have been reported in fly brains [36, 37], genetic and neural bases of sexual behaviors [38] are mapped to broad regions of the central nervous system [39, 40], suggesting neuroanatomical and functional differences between the sexes. For instance, three glomeruli are significantly larger in male fruit flies, and two of these are innervated by *fruitless* (*fru*) olfactory neurons that are required for male courtship [41, 42]. The transcription factors *dsx* and *fru* control the sexual differentiation of neural circuits and exhibit sex-specific spatial distributions in the nervous system [43, 44]. Male brains express *dsx* in 150 cells per hemisphere in 10 anatomical clusters, while female brains express *dsx* in 30–40 cells per hemisphere in 7–8 clusters [32, 45–49]. Although several neuronal clusters are not sexually dimorphic in the number of *dsx*-expressing cells, their axonal projection patterns differ between sexes [32]. The association between *fru*, *dsx* [16, 45–47, 50], and sexually dimorphic neuroanatomy, physiology, and behavior [44, 51–53], highlights the importance of studying sex-specific brain gene expression.

Sensitive high-throughput RNA-seq methodology captures variation in gene expression, which precedes other robust and subtle dimorphic phenotypes. Fruit flies have been used to study sex differences in the brain via RNA-seq, including responses to traumatic brain injury, cocaine, and developmental alcohol exposure [54–57]. Greater gene expression response to traumatic brain injury was reported in females than in males at 1, 2, and 4 h of post-injury [54], while the response among Tau-deficient individuals was greater in males [55].

Designating sex as a controlled variable to account for sex differences has been historically neglected [58, 59]. Consequently, the National Institutes of Health has emphasized sex as a biological variable (SABV); their 2015 notice [60] required researchers seeking funding to consider SABV in their studies. While recent publications utilize SABV, the confounding effects of temporal variation on sexual dimorphism in the young adult fruit fly transcriptome have not been investigated. Such effects are important because, in many species, one sex is larger and has a longer maturation time [61–64]. Although female and male *D. melanogaster* share similar molting and eclosion times [13], females take longer to reproductively mature [65]. The temporal signature of the brain transcriptome may continue to be sex-specific during early-to-middle adulthood. In this study, we characterize the female and male brain transcriptomes at three distinct adult ages to identify gene expression differences relative to sex, age, and sex-by-age interaction.

## Materials and Methods

### Fruit Fly Collection, Imaging, and Dissection

**Fig. 1:**
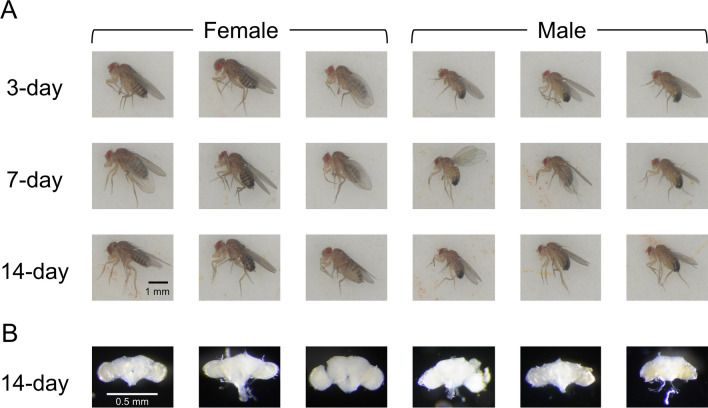
*Drosophila melanogaster* developmental comparisons between 3-, 7-, and 14-day-old female and male flies and 14-day-old brains.** A** Images of flies at 3 days (first row), 7 days (second row), and 14 days (third row) of post-eclosion. **B** Brains isolated from 14-day-old flies. Scale bars are 1 mm for whole flies and 0.5 mm for brains

Oregon wild-type *D. melanogaster* (Carolina Biological Supply Company, Burlington, North Carolina, USA) were reared in vials with standard cornmeal agar medium, under 12 h light/12 h dark cycle at 25 °C. Female and male virgin fruit flies were separated within 4 h of post-eclosion under light CO_2_ anesthesia. Flies were aged to 3, 7, or 14 days post-eclosion and snap-frozen at -80 °C. Brains were dissected in phosphate-buffered saline and stored in TRIzol to prevent RNA degradation. Three biological replicates consisting of 100 pooled brains were collected for each sex at each age. For imaging, 3-, 7-, and 14-day-old individually housed flies were anesthetized with CO_2_ and photographed with a Nikon D7100 mounted on a Leica MZ FLIII stereomicroscope with additional lighting and Camera Control Pro 2 (Nikon) imaging software (Fig. 1A). Fly brains were then dissected from 14-day flies, fixed in freshly prepared 4% paraformaldehyde, and imaged with the same camera setup (Fig. 1B).

### RNA Extraction and Sequencing

Brain RNA was isolated as described in Vu et al*.* [4]. Briefly, pooled brains (100 brains/sample) were homogenized in TRIzol (Invitrogen, Carlsbad, CA, USA) via a bead mill. Total RNA was isolated using the TRIzol reagent protocol and stored at -80 °C. RNA quantity and quality were determined with a Qubit 4 Fluorometer and Bioanalyzer 2100 (Agilent Technologies, Santa Clara, CA, USA). RNA samples were prepared for RNA sequencing using the TruSeq RNA Library Prep Kit v2 and subsequently sequenced with a NextSeq2000 Sequencing System at Scripps Research Genomics Core (La Jolla, CA, USA).

### RNA Sequencing Data Processing

Single-end sequencing reads were trimmed to remove adapter sequences via Trimmomatic (version 0.39) [66]. Sortmerna (version 2.1) was used to remove ribosomal RNA contamination [67]. Base sequences with a Phred score below 32 were removed and a minimum sequence length filter of 18 was applied. Illumina sequence reads were mapped to the reference *D. melanogaster* genome (FlyBase 6.32) [68] using STAR (version 2.7.3a) [69] with default parameters. Raw and processed files were deposited to the Gene Expression Omnibus database (accession number GSE199164).

### Differential Expression Analysis

Principal component analysis (PCA) was implemented in R (version 2.14.1) [70] and visualized with ggplot2 (version 3.4.2) [71]. The DESeq2 package (version 2.10) [72] was used to determine differential expression between sexes and ages of 13439 genes, with a significance threshold of adjusted *p* < 0.1 or unadjusted *p* < 0.05. DESeq2 filters genes to maximize results at a target false discovery rate (FDR), which is by default 0.1, as is used in *Love *et al*.* [72]. Therefore, *p* < 0.1 was used as the threshold for adjusted *p*-values; the threshold of unadjusted *p*-values was set to 0.05 to compromise between false negatives and false positives, because removing FDR correction increases the risk of false positives, while lowering the threshold decreases this risk [73–76]. Read count normalization was performed during DESeq2 analysis using the default method [72, 77–79], and log_2_(fold-changes) (LFCs) were shrunk via the ashr package (version 2.2–54) [80]. Heatmaps of LFCs with Ward’s hierarchical clustering were created using the dendextend (version 1.15.2) [81] and ComplexHeatmap (version 2.12) [82] packages in R, and Venn diagrams of differentially expressed genes (DEGs) were created with the VennDiagram (version 1.7.3) package [83]. DEGs (*p* < 0.05) by comparison and up- vs. downregulation were tested for X-chromosome enrichment using Fisher’s exact test in R, with a significance threshold of *p* < 0.05. For simplicity, experimental groups and pairwise comparisons are abbreviated as shown in Table 1. To better characterize patterns specific to any age or sex, we performed all pairwise comparisons using Wald’s test in DESeq2. While DESeq2’s likelihood ratio test can identify genes with overall differential expression over time or sex-specific expression patterns [72], pairwise Wald’s tests have been used to characterize differential expression between individual time points [84, 85].

**Table 1:** Notations of brain gene expression comparisons

Within sex	F3v7, F3v14, F7v14, M3v7, M7v14, M3v14
Between sexes	F3vM3, F3vM7, F3vM14, F7vM3, F7vM7, F7vM14, F14vM3, F14vM7, F14vM14

F (female) and M (male) indicate sex, and 3, 7, and 14 indicate post-eclosion age in days. For example, F3v7 = comparison within females, between 3 and 7 days, and F3vM3 = comparison between females and males at 3 days

### qRT-PCR Validation of RNA-Sequencing

Ten genes with a minimum between-sex LFC of 4 were selected for qRT-PCR validation: three male-biased (higher expression in males) genes at 3 days, four female-biased (higher expression in females) genes at 3 days, one female-biased gene at 14 days, and two genes with similar levels of sexually dimorphic gene expression between 3-, 7-, and 14-day flies. RNA samples were prepared using the iTaq™ Universal SYBR® Green One-Step Kit protocol. qRT-PCR was performed using Quantstudio 3 (Thermo Fisher Scientific, Waltham, Massachusetts, USA), with analysis using QuantStudio Design and Analysis (Quantstudio 3, ThermoFisher Scientific). qRT-PCR gene expression values and trimmed mean of M (TMM) RNA-sequencing counts were normalized to the housekeeping gene RpL32 (Dm02151827_g1). Correlations between resulting fold changes were performed in JMP Pro (version 14.0, SAS Institute Inc, Cary, NC, USA).

### Gene Ontology

Gene Ontology: Biological Process (GO: BP) enrichment was analyzed using the g:GOst tool of g:Profiler (Version: Ensembl 55, Ensemble genomes 55) [86], with a background consisting of detected annotated genes. Enrichment analyses were conducted on DEGs determined by significance thresholds of *p* < 0.05 and adjusted *p* < 0.1; those using adjusted *p* < 0.1 are provided as a supplementary reference (Figure S1-2). X-linked DEGs (*p* < 0.05) were analyzed with a background of detected annotated genes on the X chromosome. The top five most significant driver terms (*p* < 0.05, g:SCS corrected threshold) for each gene set were plotted in R. The GOSemSim package (version 2.24.0) [87] was used to quantify semantic similarity (Wang measure) [88] between significant GO terms (*p* < 0.05, g:SCS corrected threshold), as means between pairs of GO terms. Pairwise semantic similarity between the top five driver terms, subtracted from one, were used as distances for hierarchical clustering to create nine clusters of semantically similar terms (Figure S3, Table S1).

### Male Delayed Expression Analysis

DEGs (*p* < 0.05) in F3v7 and M7v14 comparisons were analyzed to investigate delayed expression in males. Correlations between LFCs of each gene were quantified via Pearson’s product-moment. Slopes between the ages for each sex were calculated without and with correction for number of days. Patterns of expression over time were classified as either flat (|slope|< 0.05), rising (slope > 0.05), or dropping (slope <  − 0.05), with a cutoff determined as the point between slope modes. Genes with expression patterns delayed in males were analyzed for gene ontology enrichment. Results were visualized using ggplot2 and Python (version 3.11.3) [89] with Seaborn (version 0.12.2) [90] and Matplotlib (version 3.7.1) [91] libraries.

### Transcription Factor Enrichment Analysis

Transcription factor enrichment analysis was performed on delayed DEGs (*p* < 0.05) using the RcisTarget package (version 1.20.0) [92] with the *D. melanogaster* ranking and annotation databases (flybase_r6.02 v8) [93, 94], and visualized with ggplot2. A transcription factor was considered enriched if it matched to an over-represented motif with high confidence.

## Results

Images of representative female and male flies at 3, 7, and 14 days illustrate their sexually dimorphic morphology (Fig. 1A). Female flies are larger with elongated abdomens and distinct stripes throughout. Males have dark, rounded abdomens with fewer stripes and dark spots on their front legs, known as sex combs. These differences are consistent at all three ages. Brains at 14 days have no apparent differences (Fig. 1B).

**Fig. 2:**
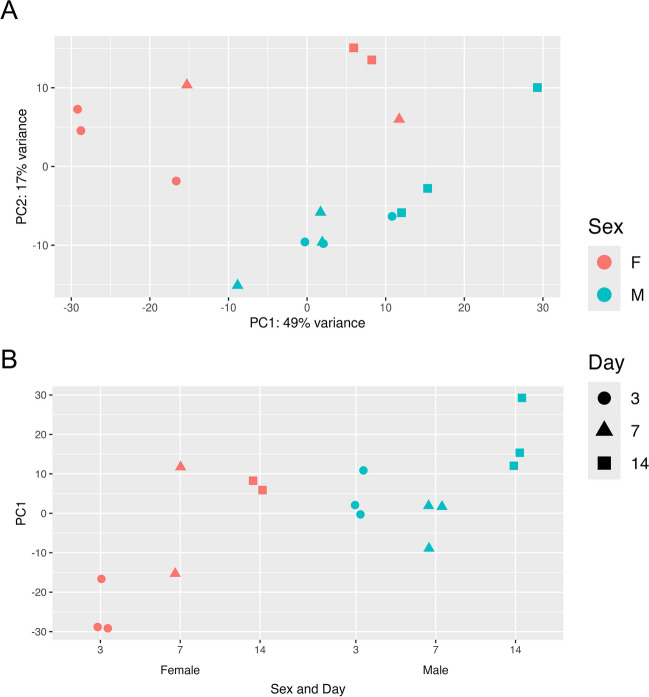
**A** Principal component analysis of gene expression profiles for each sample by sex and age after outliers are removed (*N* = 16), and **B** a scatter plot of principal component 1 (PC1) values by sex and age. One 7-day female sample and one 14-day female sample were considered outliers and removed from downstream analyses. Colors represent sexes, and shapes represent post-eclosion age in days. To avoid overlapping points in the PC1 scatterplot, each point’s *x*-axis position randomly varies within a small range centered on the “Sex and Day”

Two PCA-identified outliers (one 7-day female replicate and one 14-day female replicate) were removed from downstream analyses (Figure S4). The first two principal components explain 49% and 17% of the total variance, respectively (Fig. 2). The separation between sexes is apparent along the second principal component with a less evident clustering by age along the first principal component (Fig. 2B). Despite the range overlaps between groups, samples within the same sex and age group tend to have similar PC1 values, indicating clustering by age within each sex (Fig. 2B).

**Fig. 3:**
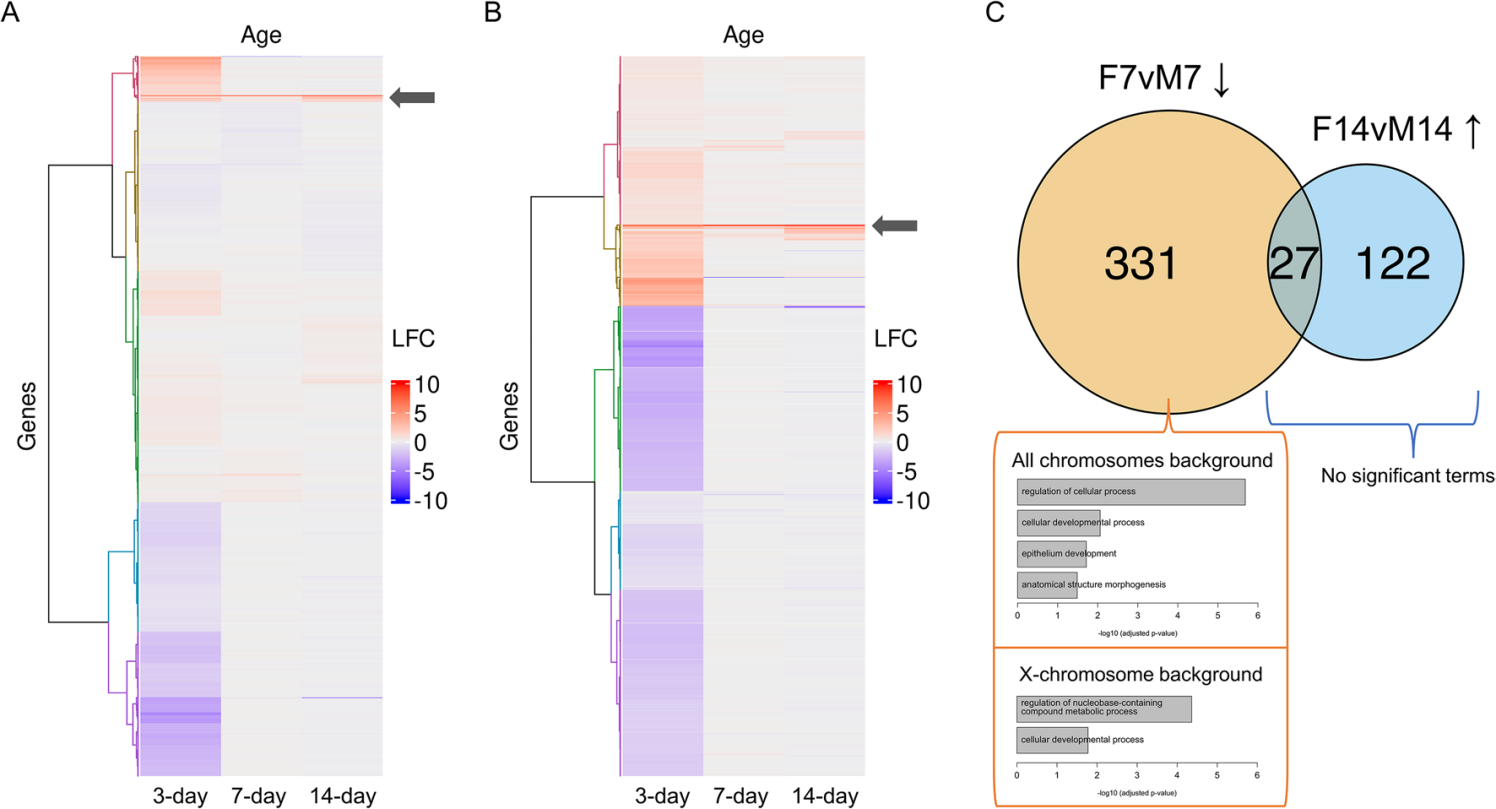
The greatest log2(fold-changes) (LFCs) between sexes are at 3 days, and female-biased genes at 7 and 14 days are overrepresented on the X chromosome. Hierarchically clustered heatmaps show genes differentially expressed between sexes at any age, **A** without FDR control (*p* < 0.05; *n* = 6698) and **B** with FDR control (adjusted *p* < 0.1; *n* = 2012), while the **C** Venn diagram quantifies X-chromosomal differentially expressed genes (*p* < 0.05) and Gene Ontology enrichment of comparisons with significant X-chromosome enrichment. Ward’s method was applied to determine clusters. Blue heatmap cells represent downregulation in females compared to males while red represents upregulation in females. Only female-biased genes at 7 days exhibited enriched GO terms (adjusted *p* < 0.05); driver terms as determined by g:Profiler are listed in the bar plots by decreasing significance, analyzed against a background of all detected annotated genes or a background limited to the X chromosome

DEGs between sexes within each age cluster by female and male bias at 3 days (Fig. 3). Of the 2012 DEGs (adjusted *p* < 0.1), 646 were female-biased and 1302 were male-biased at 3 days, while the 7-day and 14-day gene expression patterns are similar between the two sexes. Only 64 DEGs were female-biased and 18 were male-biased at 7 days, while only 73 DEGs were female-biased and 14 were male-biased at 14 days. A subset of 427 genes were consistently upregulated and 203 genes were consistently downregulated in females (Fig. 3B, Figure S5). GO analysis on a heavily female-biased cluster (LFCs between − 0.05 and 9.5) of 44 genes (Fig. 3A, B, indicated by arrows) highlighted biological functions related to defense response. The X-chromosomal genes were overrepresented in DEGs (*p* < 0.05) upregulated in females at 7 days (358 genes, *p* < 0.05) and 14 days, but not at 3 days (149 genes, *p* < 0.05; Fig. 3C). Analysis of X-chromosome enrichment was therefore only conducted on 7- and 14-day genes: GO analysis at 7 days revealed five major functional categories when considering a background of all detected annotated genes (g:GOSt adjusted *p* < 0.05 driver terms; regulation of biological process, cellular developmental process, epithelium development, anatomical structure morphogenesis) and two major categories when limiting the background to only the X chromosome (g:GOSt adjusted *p* < 0.05 driver terms; regulation of nucleobase-containing compound metabolic process, cellular developmental process). There was no significant functional enrichment at 14 days or among the 27 genes that overlap between the two ages.

**Fig. 4:**
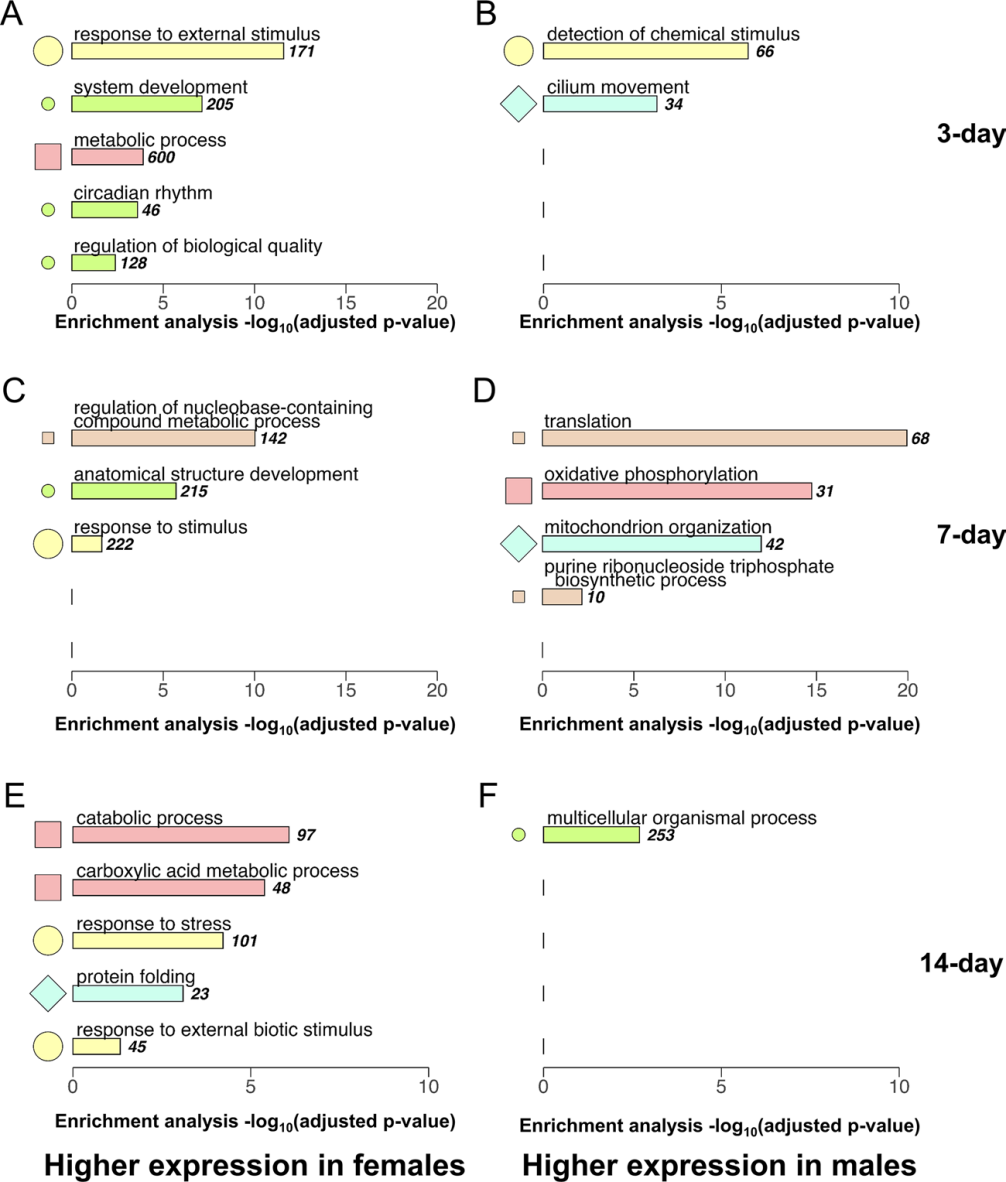
Gene ontology (GO) enrichment for differentially expressed genes (*p* < 0.05) in female vs. male flies aged (**A**, **B**) 3, (**C, D**) 7, and (**E**, **F**) 14 days. The top five significant driver GO terms (adjusted *p* < 0.05) as determined by g:Profiler are listed in decreasing significance for each gene set, with varying *x*-axis scales associated with -log_10_(adjusted *p*-values) from GO enrichment analysis. Each color and symbol represent a cluster determined by hierarchical clustering (Figure S3, Table S1) on pairwise Wang semantic similarity measures (*k* = 9). For example, “response to external stimulus” and “detection of chemical stimulus” are both in the yellow/large circle cluster and are therefore semantically similar, but “system development” is in the green/small circle cluster and is therefore not semantically similar to either yellow/large circle cluster term. The number of DEGs that enrich each GO term is displayed to the right; DEGs may enrich multiple terms in the same panel and not all DEGs enrich terms

GO terms enriched by DEGs between sexes at each age (DEGs *p* < 0.05; GO adjusted *p* < 0.05), separated by up- or downregulation in females, are presented in Fig. 4. Clusters of semantically similar GO terms are indicated by colors and symbols (Figure S3, Table S1). Genes upregulated in females at all ages enrich more terms, which more consistently belong to the same clusters. For example, females’ upregulated DEGs enriched terms related to responses to stimuli across all three ages (Fig. 4A, C, E). The most significantly enriched GO term was translation, enriched by downregulated DEGs in females at 7 days (Fig. 4D). Regulation of nucleobase-containing compound metabolic process was the term most significantly enriched by upregulated genes in 7-day females (Fig. 4C).

**Fig. 5:**
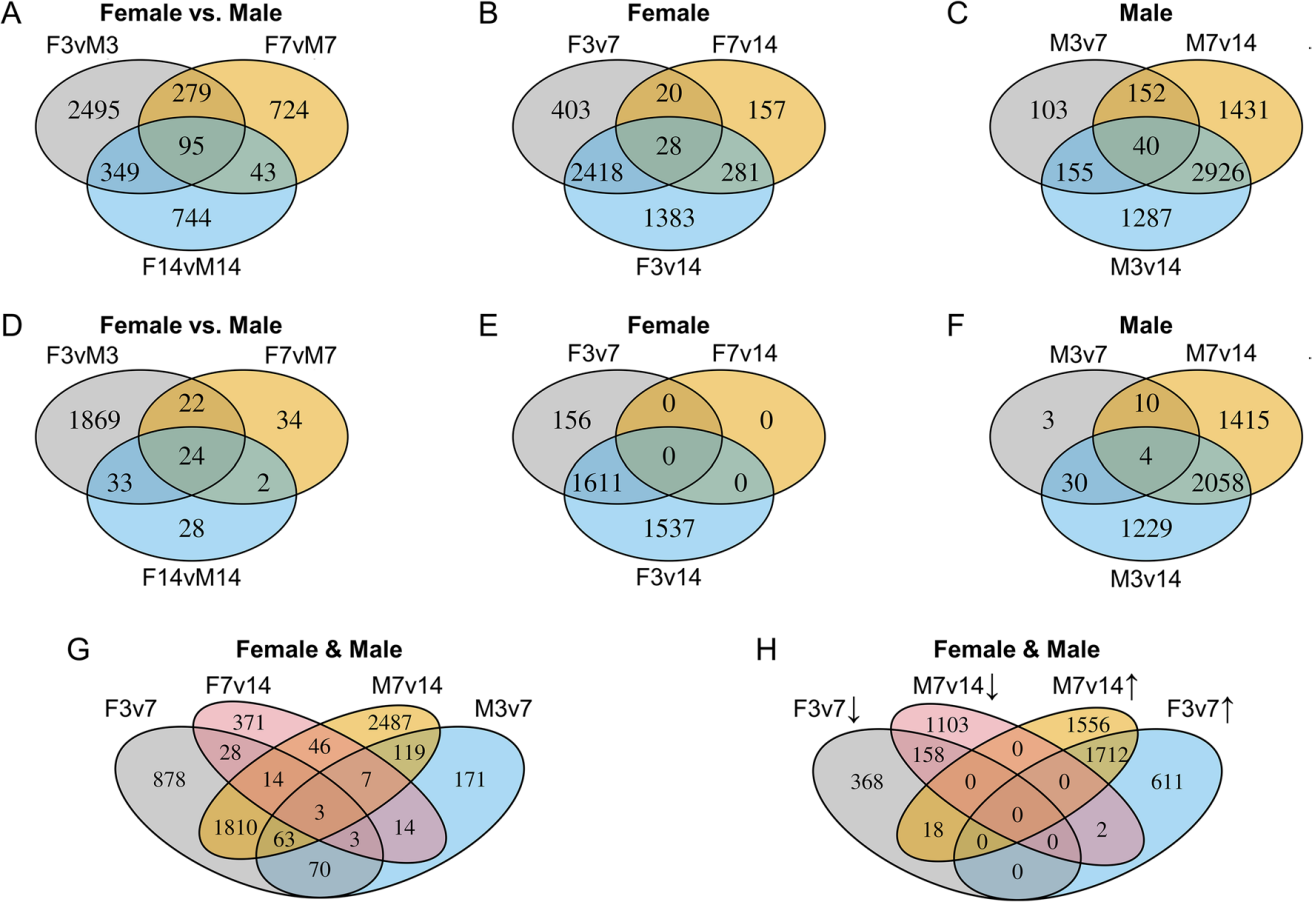
The numbers of DEGs from each pairwise comparison **A**, **D** between sexes within each age group, and **B, C**, **E**, **F** between age groups within each sex, **D**, **F** with FDR control (adjusted *p* < 0.1) and **A**, **C**, **G**, **H** without (*p* < 0.05). **G** 29.8% (1890 of 6084 DEGs) of DEGs were identified in both F3v7 and M7v14 comparisons, **H** among which 1712 are upregulated in the older flies (F7 and M14) for each sex

Female vs. male comparisons revealed the most DEGs at 3 days (3218, *p* < 0.05; 1948, adjusted *p* < 0.1), compared to the 7-day (1141, *p* < 0.05; 82, adjusted *p* < 0.1) and 14-day flies (1231, *p* < 0.05; 87, adjusted *p* < 0.1). The fewest between-sex DEGs were shared by 7-day and 14-day flies (Fig. 5A, D). In females, F3v7 and F3v14 have more DEGs than F7v14 (Fig. 5B, E), and the greatest overlap (*p* < 0.05: 2418) among age comparisons. Per this trend, 7-day females exhibit fewer differences in gene expression as they age, and younger (3-day) females show critical differences in gene activity compared to the other ages (Fig. 5B, E). In males, there are more M3v14 and M7v14 DEGs than M3v7, with the greatest DEG overlap among all male age comparisons (2926, *p* < 0.05; Fig. 5C, F). This temporal shift in the gene expression difference is highlighted by the high number of DEGs shared between F3v7 and M7v14 (29.8%, 1810 out of 6084, *p* < 0.05; Fig. 5G), with most DEGs downregulated (1712, *p* < 0.05; Fig. 5H). Both sexes show consistent results with FDR adjustment (*p* < 0.05; Fig. 5A–F).

The 1890 DEGs (*p* < 0.05) in both F3v7 and M7v14 were significantly correlated by LFC (Pearson’s correlation, *p* < 0.01; *r* = 0.57) and not significantly overrepresented on the X chromosome (Fisher’s exact test, *p* > 0.9). Of these, 1712 (91%) were upregulated in older flies of both sexes, and 158 (8%) were downregulated in both sexes (Fig. 5H). Eighteen genes downregulated only in females enriched visual perception, and the two genes downregulated only in males were *Moca-cyp* and *Zw10* (Fig. S6).

**Fig. 6:**
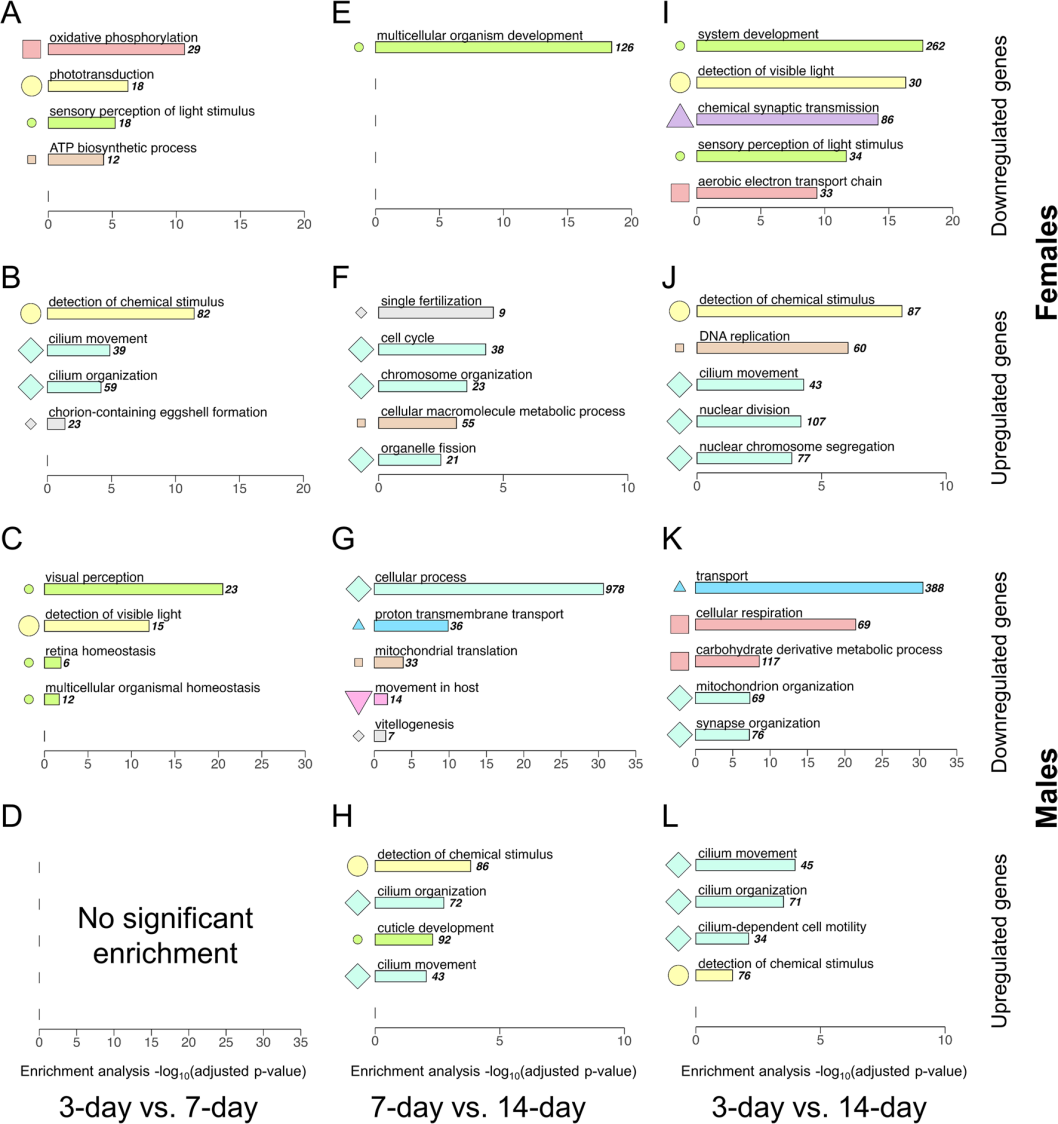
Gene ontology (GO) enrichment for differentially expressed genes (DEGs, *p* < 0.05) between 3-, 7-, or 14-day-old flies, are similar between upregulated genes in F3v7 and M7v14 comparisons. Down- and upregulations are relative to the younger flies in the comparison. Hence, 7-day flies’ genes are downregulated relative to 3-day flies’ genes. The top five significant driver GO terms (adjusted *p* < 0.05), as determined by g:Profiler, are listed in decreasing adjusted significance, with varying *x*-axis scales. Each color/symbol represents a cluster determined by hierarchical clustering (Figure S3, Table S1) on pairwise Wang semantic similarity measures (*k* = 9). For example, “phototransduction” and “detection of chemical stimulus” are both in the yellow/large circle cluster and are therefore semantically similar, but “sensory perception” is in the green/small circle cluster and is therefore not semantically similar to either yellow/large circle cluster term

GO term enrichment is similar between F3v14 and F3v7 (Fig. 6A, B, I, J), and between M3v14 and M7v14 (Fig. 6G, H, K, L), highlighting a shifted window of gene regulation between sexes. In both F3v14 and F3v7, downregulated DEGs enriched sensory perception of light stimulus, and upregulated DEGs enriched detection of chemical stimulus and cilium movement (green/small circle- and mint/large diamond-cluster terms; Fig. 6A, B, I, J). In both M3v14 and M7v14, upregulated DEGs enriched three GO terms: detection of chemical stimulus, cilium movement, and cilium organization (Fig. 6H, L). These terms are also enriched by upregulated DEGs in F3v7 (Fig. 6B, H). No other comparisons between ages within sex share the top five GO terms.

**Fig. 7:**
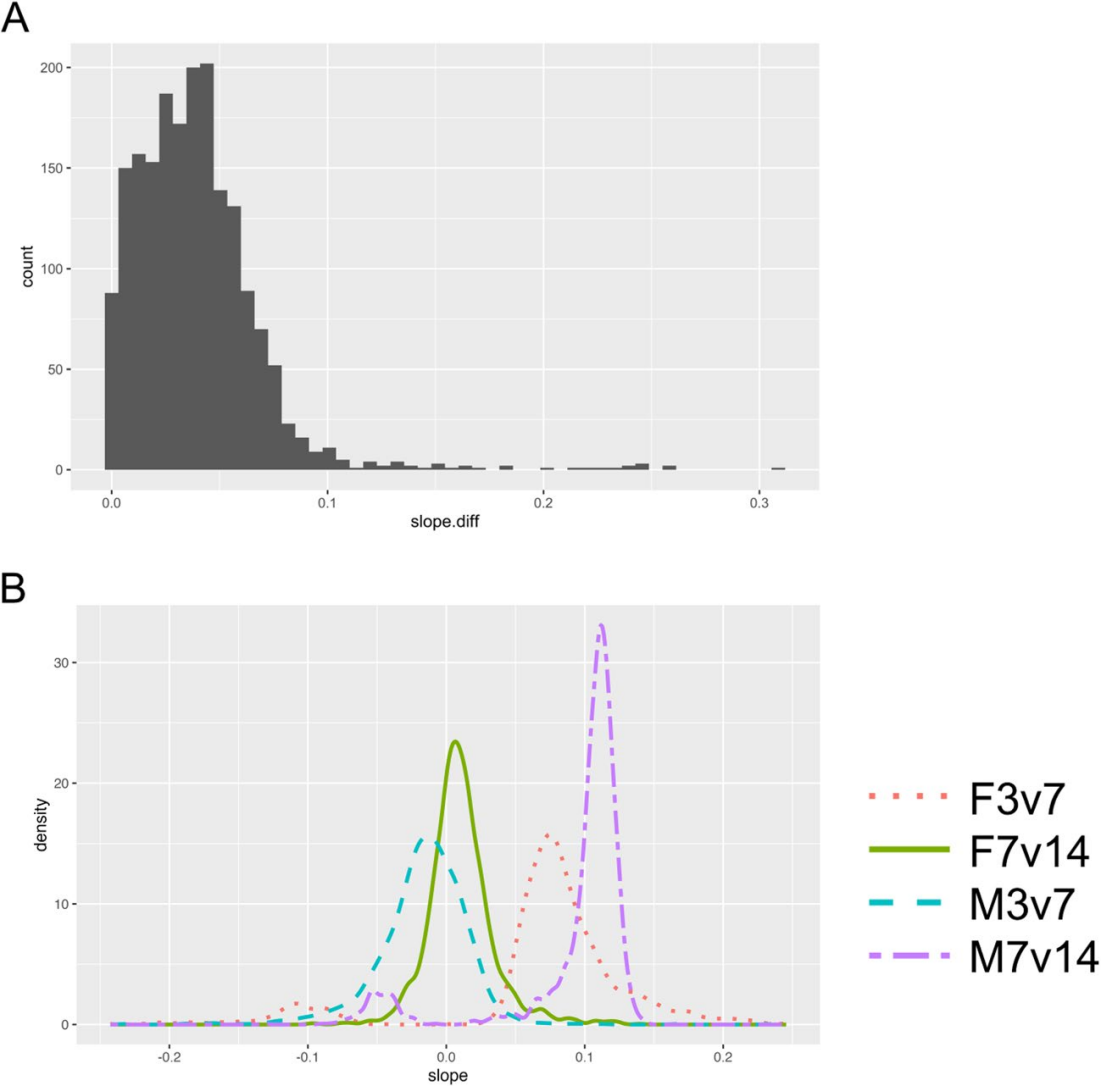
**A** Differences between 3-to-7-day and 7-to-14-day fruit flies’ gene (*p* < 0.05) expression slopes and **B** slopes by sex and time range, with normalization to a maximum of one before further correction. Slopes are divided by the number of days between ages. Colors indicate sex and time range

**Fig. 8:**
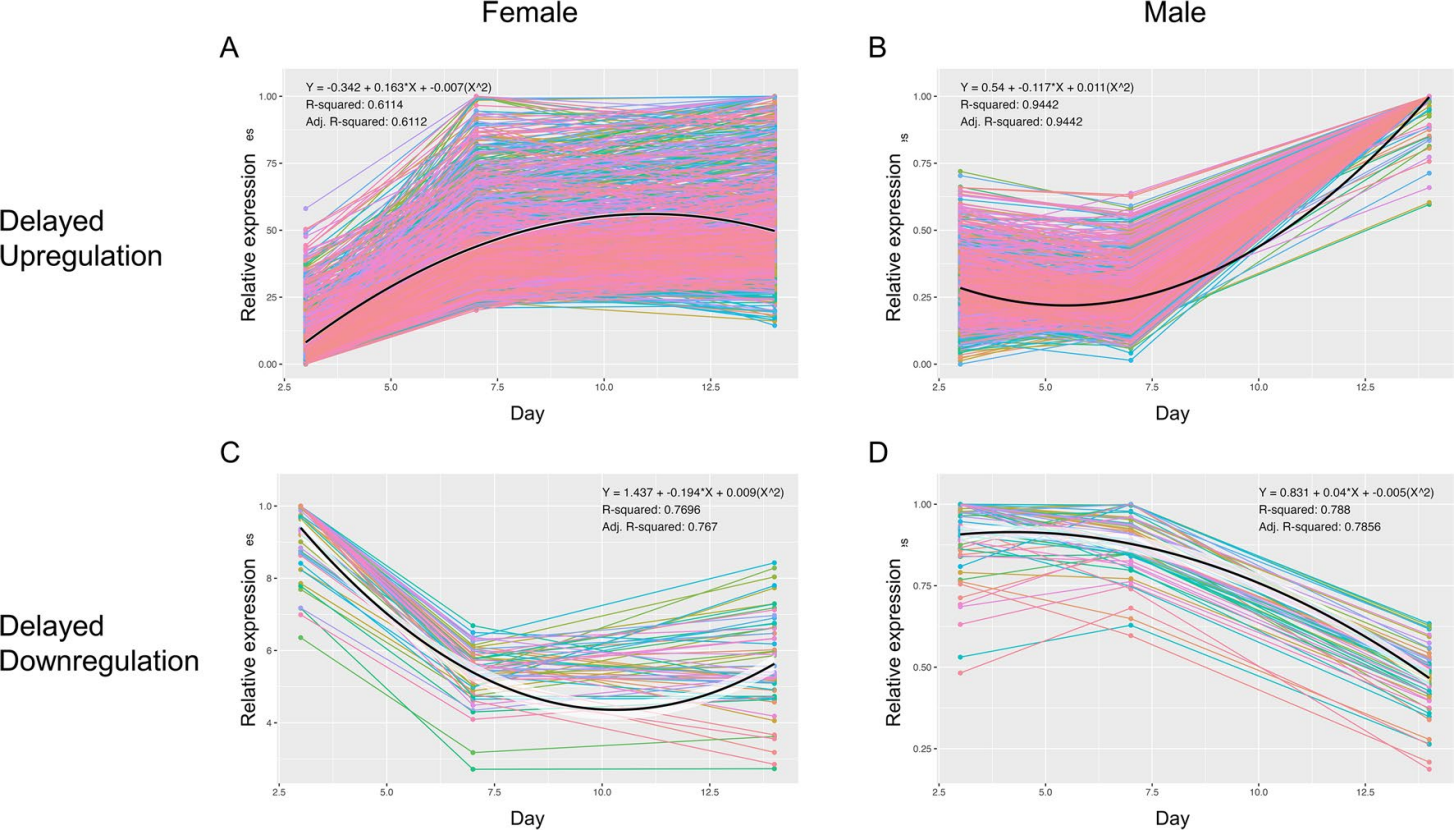
Gene expression over time for **A**, **C** female and **B**, **D** male fruit flies suggests a delay in male genes that **A**, **B** increase or **C**, **D** decrease in expression over time. Normalized mean reads are on the *y*-axis and the age is on the *x*-axis. Each line represents a gene with delayed upregulation or downregulation patterns, and line colors are arbitrary

**Fig. 9:**
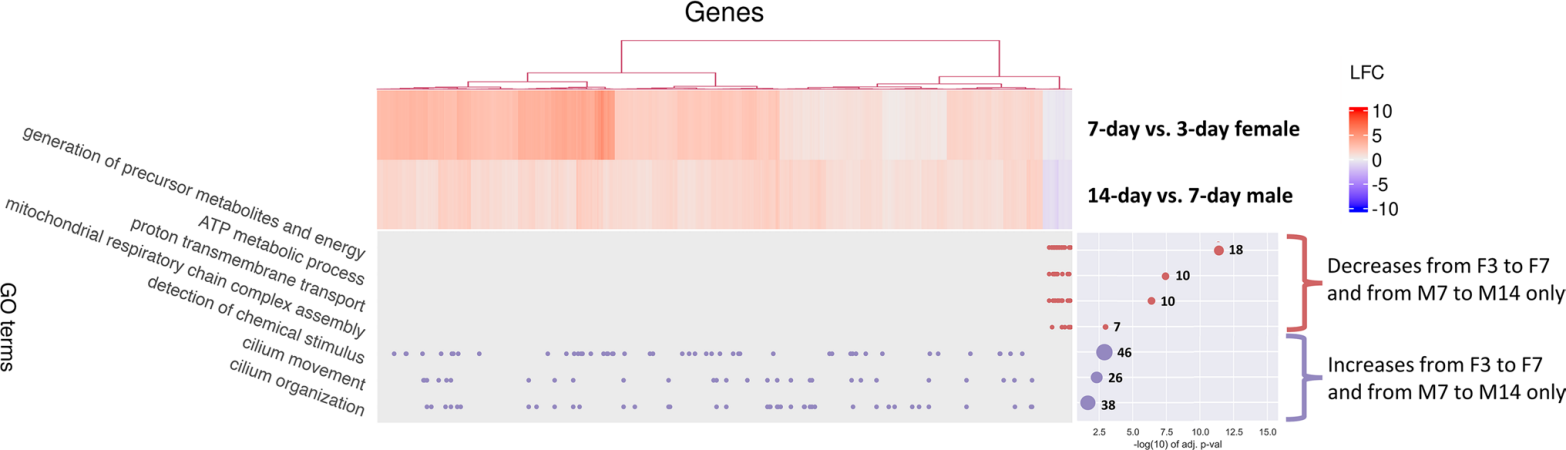
Of the 1890 DEGs (*p* < 0.05) identified in F3v7 and M7v14 comparisons, 1870 (98.9%) show similar gene regulation patterns between the older and the younger flies. These 1870 genes are presented in the heatmap and hierarchical clustering with relative gene expression levels in F3v7 and M7v14 comparisons. In the GO plot below the heatmap, each point represents an association between a gene and an enriched term. Log2(fold-change) (LFC) represents the later *vs*. the earlier age, so genes with positive LFC increased in expression over time. Red cell colors depict upregulation over time, and blue colors depict downregulation over time. Bubbles of varying sizes on the right of the GO plot indicate -log10(adjusted *p*-value) of enrichment on the *x*-axis and the number of genes contributing to that GO term’s enrichment by size and value label. Therefore, larger bubbles indicate more term-associated genes in the gene set, with exact values presented as labels next to the bubbles

Time-corrected slopes of DEG (*p* < 0.05) expression in F3v7 and M7v14, but not in F7v14 and M3v7, are shown in Fig. 7. Differences between 3-to-7-day and 7-to-14-day slopes were skewed right with a mode around 0.5 before accounting for the time difference between comparisons (Fig. S7A). Correcting for time (dividing the slopes by the range of days) skewed the overall slope differences further, decreasing the mode to just below 0.05 (Fig. S7A). Regardless of correction, the modes of M3v7 and F7v14 slopes ranged from approximately -0.01 to -0.05, while the correction decreased the difference in modes between F3v7 and M7v14 slopes from approximately 0.5 to 0.04 (Fig. S7B, Fig. 7B). Considering normalized and corrected slopes, the expression of 1548 genes in females increased (slope > 0.05) from 3 to 7 days and was stable (|slope| < 0.05) from 7 to 14 days, while the expression of 1496 genes in males was stable from 3 to 7 days and increased from 7 to 14 days (Fig. 8). The overlapping 1361 genes suggest delayed upregulation in males and enrich for detection of chemical stimulus, and cilium movement and organization (Fig. 9). In females, the expression of 173 genes decreased (slope < -0.05) from 3 to 7 days but remained stable from 7 to 14 days; this pattern is mirrored but delayed in all 61 genes that were stable in males from 3 to 7 days then decreased from 7 to 14 days (Fig. 8), strongly suggesting delayed downregulation. These genes were enriched for ATP metabolic process, proton transmembrane transport, and mitochondrial respiratory chain complex assembly (Fig. 9). Hierarchical clustering of both delayed upregulated and downregulated genes reveals two main clusters: genes with greater LFC in F3v7 than M7v14 (red cluster), and genes with relatively similar LFCs in F3v7 and M7v14 (blue cluster; Fig. 9).

Among the 61 genes with delayed downregulation in males, 47 significantly enrich 44 transcription factors (TFs) (Figure S8); four TFs, v*ri*, *Pdp1*, *gt*, and *CG7786*, are associated with over 68% of these genes; *Sox 14*, *21a*, and *102f* are enriched by both delayed-upregulated genes and delayed-downregulated genes (Figure S8B). Most of the enriched TFs, including the three *Sox* genes, are involved in development and cell differentiation.

Ten genes, *SA-2*, *SOLO*, CG10182, *Yp1*, *Yp3*, *Acp70A*, CG43055, *Jon65Aiv*, CG5107, and CG13428, were selected to validate RNA-sequencing results using qRT-PCR. After normalization to the housekeeping gene, *RpL32*, RNA-sequencing and qRT-PCR showed similar expression profiles and correlate well (adjusted *R*^2^ = 0.918, *p* < 0.0001) (Figure S9). While expression levels vary between the two methods, higher vs. lower expressions relative to the housekeeping gene are consistent, except for *Jon65Aiv* in 3-day female flies, showing a higher expression level in qRT-PCR but lower in RNA-sequencing (Figure S9).

## Discussion

Per physiological and morphological dimorphism (Fig. 1), and delayed maturation between sexes in many species [29–31] including fruit flies [27, 28], we hypothesized that female and male fruit fly brains exhibit signatures of delayed gene expression during early-to-middle adulthood. We used RNA-Seq to quantify the brain transcriptome at three distinct adult ages: 3, 7, and 14 days of post-eclosion. We identified sex, age, and sex-by-age gene expression signatures, which often precede more observable morphological and physiological variations and indicate subtle brain dimorphisms [95–98]. We detected 6698 DEGs between sexes within the same age, with the most divergent expression at 3 days. Between ages, 6084 DEGs were detected, with 1890 sharing similar expression changes from 3 to 7 days in females, and from 7 to 14 days in males. Most of them (1712, 90.5%) were upregulated and enriched for chemical stimulus detection and/or cilium regulation. This subset of DEGs highlights a temporal shift in the brain gene regulation between females and males comprising over 10% of tested genes.

While sex-biases were present at all ages, 23.4% of DEGs exhibited a delay that accounts for some of the observed sex-bias within each age. Notably, while most of our analysis is based on unadjusted *p* < 0.05 threshold, we present both FDR-corrected and uncorrected data to achieve two goals. The adjusted *p*-value threshold is more conservative, guarding against false positives to give an understanding of which specific genes’ expression levels are most likely affected by sex and age. Relaxing the threshold by removing FDR correction allows us to minimize false negatives at the risk of false positives and understand the overall data patterns. We placed greater emphasis on a hypothesis that requires a larger pool of candidate genes. Due to the relatively short age ranges, we expected difficulty detecting many subtle changes in gene expression without relaxing significance criteria. Adjusted *p*-values are important at the individual gene level, while unadjusted *p*-values can detect broad patterns overlooked by more conservative, adjusted *p*-values.

Throughout the lifespan, many sexually reproducing species maintain sexually dimorphic phenotypes including size, morphological and anatomical features (Fig. 1) [13, 99, 100], reproductive commitment [27, 101], and behavior [47, 52, 53, 102, 103]. Significant anatomical, morphological, neural, and gene expression differences between female and male fly brains are well-documented [16, 37, 44, 46, 51, 104]. The body size of fruit flies, limited by the exoskeleton, does not change post-hatching, and on average, female flies remain larger than males, with both sexes displaying morphological differences (Fig. 1) [13, 99, 105, 106]. Although the anatomical and morphological changes are not obvious during adulthood, subtle phenotypic differences both within and between sexes should be quantifiable at the gene expression level, particularly in stimulus-responsive tissue such as the brain [104]. To better understand the brain gene activity relevant to sexually dimorphic phenotypes of early and mid-adulthood, we compared brain transcriptomes of 3-, 7-, and 14-day fruit flies.

The clear spatial distinction between sexes by PCA, mostly across the PC2 axis, suggests a major effect of sex on the brain transcriptomic profile across all three ages (Fig. 2). In fruit flies, the body size difference (Fig. 1) is controlled by the expression of *tra*, a sex-determining gene, and the dosage of *Myc*, an X-chromosomal gene [99]; in humans, sex-biased genes explain 12% of height differences [107]. Sexually dimorphic gene expression is found in both fruit flies and mammals, although the extent varies between species, tissue, and age [107–110]. An overabundance of sex-biased genes on the X chromosome in fruit fly brains possibly due to dosage compensation in males reported by *Catalán *et al*.* [110], is consistent with the majority of female-biased DEGs at 7 and 14 days (Fig. 3C). Age groups span mostly along the PC1 axis (Fig. 2). The higher explained variation of PC1 (49%) than PC2 (17%) highlights more robust gene expression differences across the three ages (Fig. 5B-C) than between sexes (Fig. 5A). The shorter distance between the two sexes in 7-day and 14-day flies on the PCA plot (Fig. 2) suggests diminishing differences over time. Indeed, throughout development, the transcriptomic landscape readily shifts and becomes less sexually dimorphic (Fig. 3A-B) [111]. *Arbeitman *et al*.* demonstrated that expression levels changed for 2103 genes during fruit fly embryogenesis and only 118 genes in adulthood [109]. Our data of early adulthood stages before 14 days still display distinct transcriptomic profiles over time, particularly in females not displaying the 3- and 7-day spatial overlap for males (Fig. 2A). Sex and life stage interact to form unique patterns of gene activity over time; female-biased transcripts increase in the first 24 h of adulthood while male-biased transcripts increase from larva to pupa stages [109]. As predicted from observed morphological differences, brain gene expression distinguishes both sex and age in fruit flies, with the most differences between the two sexes at 3 days (Figs. 2 and 3).

Fully mature oocytes in females appear at 24 h of post-eclosion, with maturation continuing past 3 days [112]. While the rate of sexual maturation varies, developing young adult fruit flies are generally fully mature and start mating no later than 3 days of post-eclosion [102, 103, 113, 114]. Throughout adulthood, males’ accessory glands grow [115], and metabolic activity between sexes becomes more dimorphic [116]. Females’ higher resting metabolic rates [117] may be implicated in the between-sex DEGs that enriched metabolic GO terms at every age, and in the 7-day X-chromosomal DEGs involved in the regulation of nucleobase-containing compound metabolic processes (Figs. 3C and 4). The upregulation of development and metabolism genes in females (Figs. 3C and 4A) may be affected by sex maturation [103] regulated by the brain at the neuronal and molecular level [102, 103]. Not surprisingly, the timing of sexual maturation by about 3 days [103], also coincides with the observed male-biased enrichment of cilium movement (Fig. 4B), which is related to spermatogenesis[118, 119].

Fruit flies exhibit sex-specific behavior [33, 41, 47, 51–53, 102, 103]. Virgin females are more active than males during the day, but less so in the morning and evening [117]. Since locomotion is driven by sensory stimulus and circadian rhythm [117, 120–122], females’ upregulated stimulus–response genes at all ages and circadian response genes at 3 days is not surprising (Figs. 3A-B and 4A). Three of the four annotated optic nerve genes, *Appl*, *RapGAP1*, and *tutl* [123–125], are also significantly downregulated at 14 days compared to 3 days in at least one sex, consistent with enriched visual perception terms in these comparisons (Fig. 5). Aside from sex, mating status has also been shown to affect chemical sensory [126], baseline behavior [127], and the neuronal regulation of behavioral responses to stressors [101]. Our fruit flies were separated by sex post-eclosion, and the gene expression profile is representative of virgin flies.

Almost 30% of the within-sex DEGs (1890 of 6084; Fig. 5B, C, E, F) were identified in both F3v7 and M7v14 comparisons, suggesting a delayed transcriptomic shift in males: expression of 1712 genes increased first in females and then in males, while 158 decreased in the same order. Many species exhibit sexually dimorphic time to maturation, implying delayed anatomical and behavioral changes in one sex [27–31]. Delayed phenotypic changes can vary as female fruit flies may undergo a change earlier than males for some phenotypes but not others. For instance, although females become hyperactive sooner post-eclosion, they start mating later than males [65, 102].GO analysis also suggests a delayed transcriptomic pattern in males, as F3v7 and M7v14 genes upregulated over time have similar functional enrichment. Across the three ages, upregulated genes enriched the detection of chemical stimulus and cilium organization/movement, mostly driven by the change between 3 to 7 days in females, but 7 to 14 days in males (Fig. 6B, H, J, L; Fig. 9). Consequently, only F3vM3 and not F7vM7 male-biased genes enrich cilium movement (Fig. 4B, D). The cilium is involved in various biological functions, including sensation and signal transduction [128–134]. Fruit flies’ sensory neuron cilia facilitate signal transduction via ion channels [135–137]; one of the shared upregulated genes, *TrpA1*, is a well-studied ciliary cation channel involved in thermosensation and chemosensation [138–142]. Females have more fibers than males within the mushroom body, a brain region responsible for olfactory learning and memory [143, 144]. The number of fibers in females grows rapidly from eclosion to 7 days and plateaus around 14 days [145], mirroring the sensory genes’ upregulation we detected during the same period (Fig. 6B, H, J, L; Fig. 9). Since we did not evaluate a relationship between the mushroom body and the delayed upregulation, additional brain anatomical evidence may clarify the observed delayed gene upregulation in male flies.

The 3v14 comparisons, encompassing both 3v7 and 7v14 analysis, are a reference point for overall expression changes. Per DEG and functional enrichment results, F3v7 and F3v14 are most similar, highlighting the relevance of earlier ages to overall transcriptomic shift in females. Conversely, the similarities between M7v14 and M3v14 emphasize the effects of later ages on male brains’ gene activity. In these comparisons, metabolic genes involved in ATP production are downregulated at a later age. Many GO terms enriched by downregulated genes in 61-day-old male flies were identified in 9- to 10-day-old males [146]. Frut flies’ ATP synthesis peaks between 18 and 40 days, declining afterwards [147–149]. While ATP levels are significantly reduced by 43–47 days compared to 1–2 days in both sexes, females’ decrease begins earlier than in males’ [150]. This may be preceded by the downregulation of related genes in the brain between 3 and 14 days, which begins earlier in females.

Both F3v14 and F7v14 DEGs significantly enriched GO terms involved in the development and the cell cycle (Fig. 6F, J). Multicellular organism and system development genes were downregulated, while DNA replication and cell cycle genes were upregulated. Considering the link between cell cycle activation and neurodegeneration [151], the exclusive downregulation of these genes in females developing and aging sooner than males. It would be interesting to test if similar downregulation patterns occur in males soon after. Contributors to females’ downregulation could be genes related to ecdysone, a steroid that regulates metamorphosis and development in larvae and pupae, and learning, memory, behavior, and circadian rhythm in adult brains [152]. Females experience a greater decrease and fluctuation in ecdysone equivalents post-eclosion [153, 154], consistent with the downregulation in developmental genes in only females. Specifically, ecdysone-related downregulated genes in F3v14 and F7v14 comparisons include ecdysone receptor (*EcR*), ecdysone-induced protein 63E (*Eip63E*), diabetes and obesity regulated (*DOR*), and taiman (*tai*). Besides the top five GO terms related to chromosomes and the cell cycle (Fig. 6), meiotic cell cycle and female gamete generation were also significantly enriched among upregulated genes in F3v14 and F7v14. This upregulation implicates oocyte generation and maturation controlled by the brain [112, 155], which varies with age: the number of ovarioles decreases 1–4 days of post eclosion [156] and increases in the next 4 days [157]. The average oocyte maturation stage decreases 4–16 days of post-eclosion [157]. This is a female-specific process expectedly lacking in the male temporal delay marked by other functional enrichments.

Analysis of normalized gene expression slopes, corrected for the difference in time from 3 to 7 days (4-day range) and from 7 to 14 days (7-day range), was used to identify genes with a similar but delayed expression change in males compared to females (Fig. 7). After selecting DEGs from only F3v7 and M7v14 comparisons, distributions overlapped between F7v14 and M3v7 slopes, and between F3v7 and M7v14 positive slopes. These overlaps are consistent with a similar albeit delayed increase in expression (Fig. 7B). The F7v14 and M3v7 slopes center around zero, confirming a late plateau in expression for females and an early plateau for males, as expected from the lack of differential expression at these ages (Figs. 7B and 8). The overlapping positive F3v7 and M7v14 slopes indicate a similar upregulation among younger females and older males (Figs. 7B and 8A, B). The negative F3v7 and M7v14 slopes overlap very little after time range correction, suggesting that the delayed decrease in expression is more extreme in younger females than older males (Fig. S7B; Figs. 7B and 8C, D).

GO analysis on genes identified via expression slopes (1422 genes; Fig. 9) is thematically consistent with the functions of DEGs between sexes (Fig. 6), indicating the significance of delayed genes in overall functional enrichment. Stimulus detection genes are overrepresented among upregulated DEGs in the F3v7 and M7v14 comparisons (Fig. 6) and among genes suggesting male-delayed upregulation (Fig. 9). This temporally shifted gene activity could explain why stimulus response genes were consistently upregulated in females when we compared the two sexes at the same age (Fig. 4). Thus, age-sex interaction should be considered when studying sensory functions in fruit flies. Similarly, ATP synthesis genes are overrepresented among DEGs with delayed downregulation in males (Figs. 6 and 9), consistent with the upregulation of oxidative phosphorylation genes in males relative to females at 7 days while metabolism genes are generally upregulated in females (Fig. 4). A delay in male development and aging is supported by the association of 7% of upregulation-delayed genes with related *Sox* TFs (Figure S8). Moreover, 13% of TFs enriched by downregulation-delayed genes are involved in development and differentiation (Figure S8).

Per morphological and physiological dimorphism, and delayed maturation between sexes in many species, we hypothesized that fly brains exhibit underlying sex-specific signatures of gene expression, which are temporal and maintained at three distinct ages in early-to-middle adulthood. Using both adjusted *p* < 0.1 and unadjusted *p* < 0.05 thresholds, we identified overall expression patterns and specific DEGs between the sexes and ages. Our data highlight an important and consistent male-specific temporal delay in gene expression. Because male-delayed gene expression patterns could contribute to between-sex comparisons at the same age, sexual dimorphism studied at physiologically comparable life stages rather than chronological age should be more biologically relevant. This issue has been extensively discussed in human development and aging studies [158–161] but is often overlooked in animal models, including fruit flies [46, 93, 104, 110, 111, 117]. Using survival ratios to determine comparable ages across populations [162] could help mitigate such confounding effects.

We recommend that studies utilize a more targeted design to quantify a broader set of post-transcriptional phenotypes relative to sex- and age-specific temporal variation. For instance, proteomic and metabolomic evidence of stimulus response and ATP metabolism delay would help determine if the observed shift in gene expression is physiologically significant. As we only assessed brain gene expression at 3, 7, and 14 days, comparing transcription at more frequent intervals would potentially uncover other temporal shifts in regulatory activities and more precisely determine temporal variations, including onsets, peaks, and cessations of physiologically relevant gene expression phenotypes. Mating status in terms of frequency and the number of competitors and potential mates, has sex- and age-specific effects on transcriptomic maturity rates [163, 164]. Gene expression in fruit fly heads varies from hours to days after mating [165, 166]. Metabolism and stimuli detection, which were enriched by the observed temporally shifted gene regulation in virgin flies, are implicated in these dynamic regulatory activities (Fig. 9). Fruit fly studies that involve mating should therefore rely on experiment-specific reference conditions to properly account for relevant effects.

## Supplementary Information

Below is the link to the electronic supplementary material.

Supplementary file1 (DOCX 11726 KB)

## Data Availability

Raw and processed RNA sequencing data have been deposited to the National Center for Biotechnology Information Gene Expression Omnibus database (accession number GSE199164).
