# Supplementary material for: Lagging Brain Gene Expression Patterns of Drosophila melanogaster Young Adult Males Confound Comparisons Between Sexes
Source: Mol Neurobiol. 2024 Aug 28;62(3):2955–72. doi: 10.1007/s12035-024-04427-7 (PMC11790743; doi:10.1007/s12035-024-04427-7)
Supplement: Supplementary file 1 — Supplementary file1 (DOCX 11726 KB) [file 12035_2024_4427_MOESM1_ESM.docx]

**Supplementary Material**

**Article Title:**

Lagging brain gene expression patterns of *Drosophila melanogaster* young adult males confound comparisons between sexes

**Authors:**

Flannery McLamb^1,2^, Zuying Feng^1^, Jeanne P. Vu^1,3^, Lindsey Griffin^1,2^, Miguel F. Vasquez^1,4^, Goran Bozinovic^1,3,5,6^

**Affiliations:**

^1^ Boz Life Science Research and Teaching Institute, San Diego, CA, USA

^2^ University of California San Diego, Division of Extended Studies, La Jolla, CA, USA

^3^ San Diego State University, Graduate School of Public Health, San Diego, CA, USA

^4^ University of California San Diego, National Center for Microscopy and Imaging Research, La Jolla, CA, USA

^5^ Portland State University, Center for Life in Extreme Environments, Portland, OR, USA

^6^ University of California San Diego, School of Biological Sciences, La Jolla, CA, USA

**Corresponding Author:** Goran Bozinovic

University of California San Diego

[gbozinovic@ucsd.edu](mailto:gbozinovic@ucsd.edu)

**Table S1. Semantic similarity clusters of GO terms from Figures 4 and 6, as determined by hierarchical clustering of Wang measure semantic similarity values subtracted from one (Figure S1)**

| **Color/Shape** | **Cluster Terms** |
| --- | --- |
| Brown/Small square | regulation of nucleobase-containing compound metabolic process, translation, purine ribonucleoside triphosphate biosynthetic process, DNA replication, cellular macromolecule metabolic process, ATP biosynthetic process, mitochondrial translation |
| Red/Large square | metabolic process, catabolic process, carboxylic acid metabolic process, oxidative phosphorylation, aerobic electron transport chain, cellular respiration, carbohydrate derivative metabolic process |
| Pink/Inverted large triangle | movement in host |
| Yellow/Large circle | response to external stimulus, response to stimulus, response to stress, response to external biotic stimulus, detection of chemical stimulus, detection of visible light, phototransduction |
| Blue/Small triangle | transport, proton transmembrane transport |
| Purple/Large triangle | chemical synaptic transmission |
| Mint/Large diamond | protein folding, cilium movement, mitochondrion organization, nuclear division, nuclear chromosome segregation, cilium organization, cell cycle, chromosome organization, organelle fission, cilium-dependent cell motility, synapse organization, cellular process |
| Gray/Small diamond | chorion-containing eggshell formation, single fertilization, vitellogenesis |
| Green/Small circle | system development, circadian rhythm, regulation of biological quality, anatomical structure development, multicellular organismal process, sensory perception of light stimulus, multicellular organism development, cuticle development, visual perception, retina homeostasis, multicellular organismal homeostasis |

**
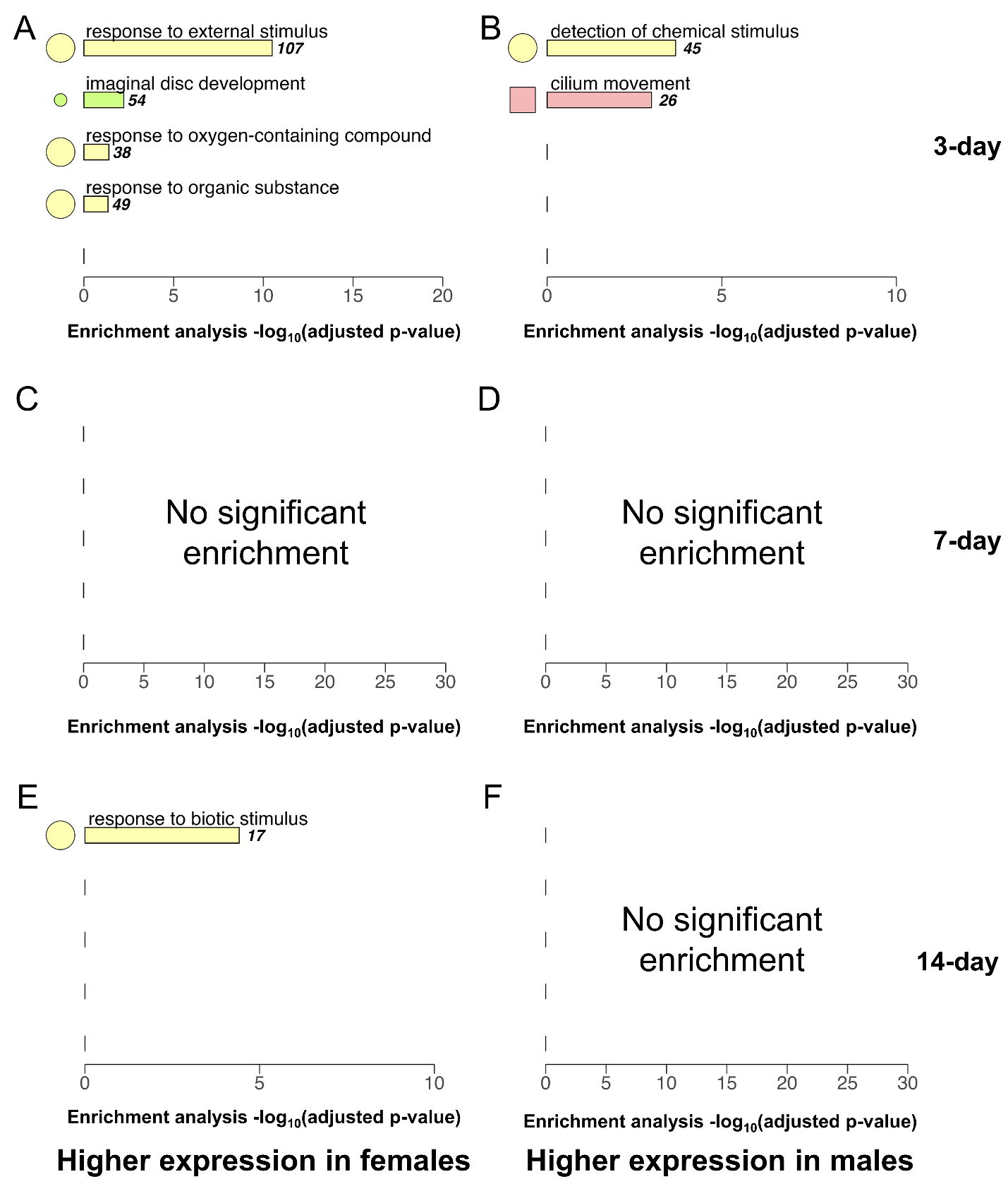
**

**Fig. S1** Gene Ontology (GO) enrichment for differentially expressed genes determined at the adjusted p < 0.1 threshold in female vs. male flies aged (A-B) 3, (C-D) 7, and (E-F) 14 days. The top five significant driver GO terms (adjusted p < 0.05) as determined by g:Profiler are listed in decreasing significance for each gene set, with varying x-axis scales associated with -log10(adjusted p-values) from GO enrichment analysis. Each color/symbol represents a cluster determined by hierarchical clustering (Figure S1, Table S1) on pairwise Wang semantic similarity measures (k = 9). For example, “response to external stimulus” and “detection of chemical stimulus” are both in the yellow/large circle cluster and are therefore semantically similar, but “system development” is in the green/small circle cluster and is therefore not semantically similar to either yellow/large circle cluster term. The number of DEGs that enrich each GO term is displayed to the right; DEGs may enrich multiple terms in the same panel and not all DEGs enrich terms

**
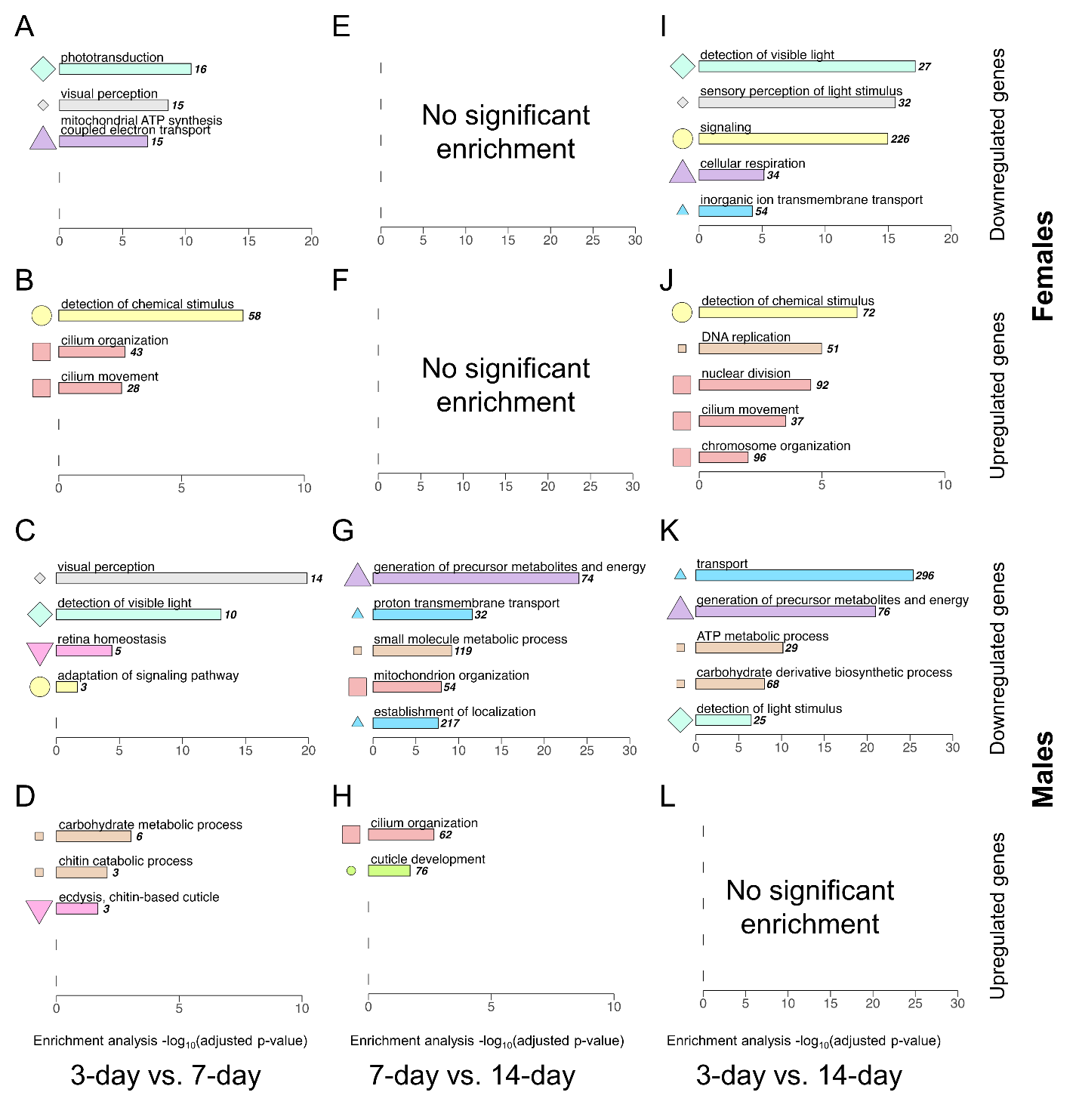
**

**Fig. S2** Gene Ontology (GO) enrichment for differentially expressed genes determined at the adjusted p < 0.1 threshold between 3-, 7-, or 14-day-old flies, are similar between upregulated genes in F3v7 and M7v14 comparisons. Down- and up-regulations are relative to the younger flies in the comparison. Hence, 7-day flies' genes are downregulated relative to 3-day flies' genes. The top five significant driver GO terms (adjusted p < 0.05), as determined by g:Profiler, are listed in decreasing adjusted significance, with varying x-axis scales. Each color/symbol represents a cluster determined by hierarchical clustering (Figure S1, Table S1) on pairwise Wang semantic similarity measures (k = 9). For example, “phototransduction” and “detection of chemical stimulus” are both in the yellow/large circle cluster and are therefore semantically similar, but “sensory perception” is in the green/small circle cluster and is therefore not semantically similar to either yellow/large circle cluster term


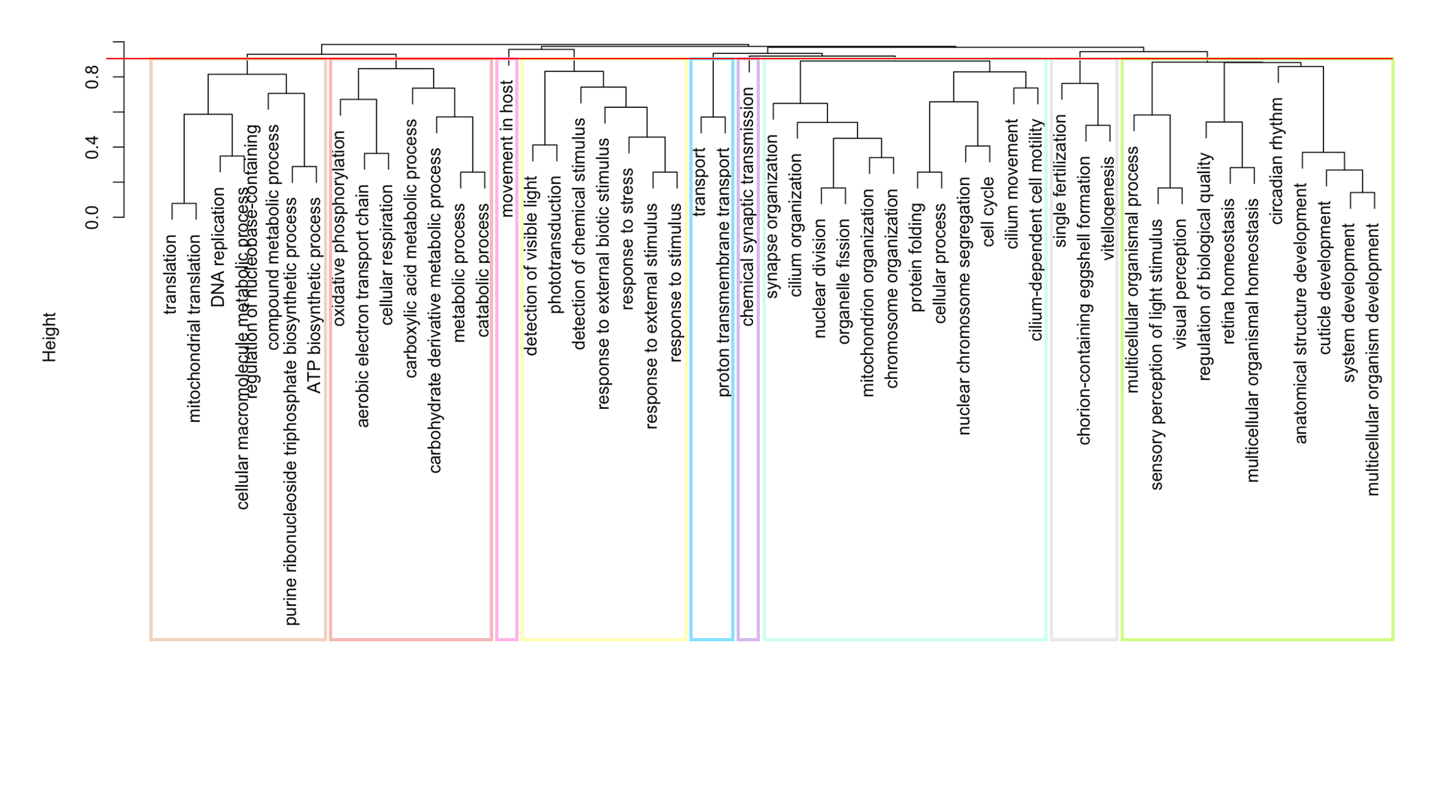


**Fig. S3** Hierarchical clustering of top five driver terms from Figures 4 and 6 gene sets by semantic similarity. Wang measure semantic similarity values subtracted from one were used as distances for clustering, so similar terms are clustered together. Longer branch length along the y-axis indicates greater dissimilarity. The bright red line is the similarity cutoff for clusters (k = 9 clusters), and the resulting clusters are boxed in the same colors used in Figures 4 and 6​

 
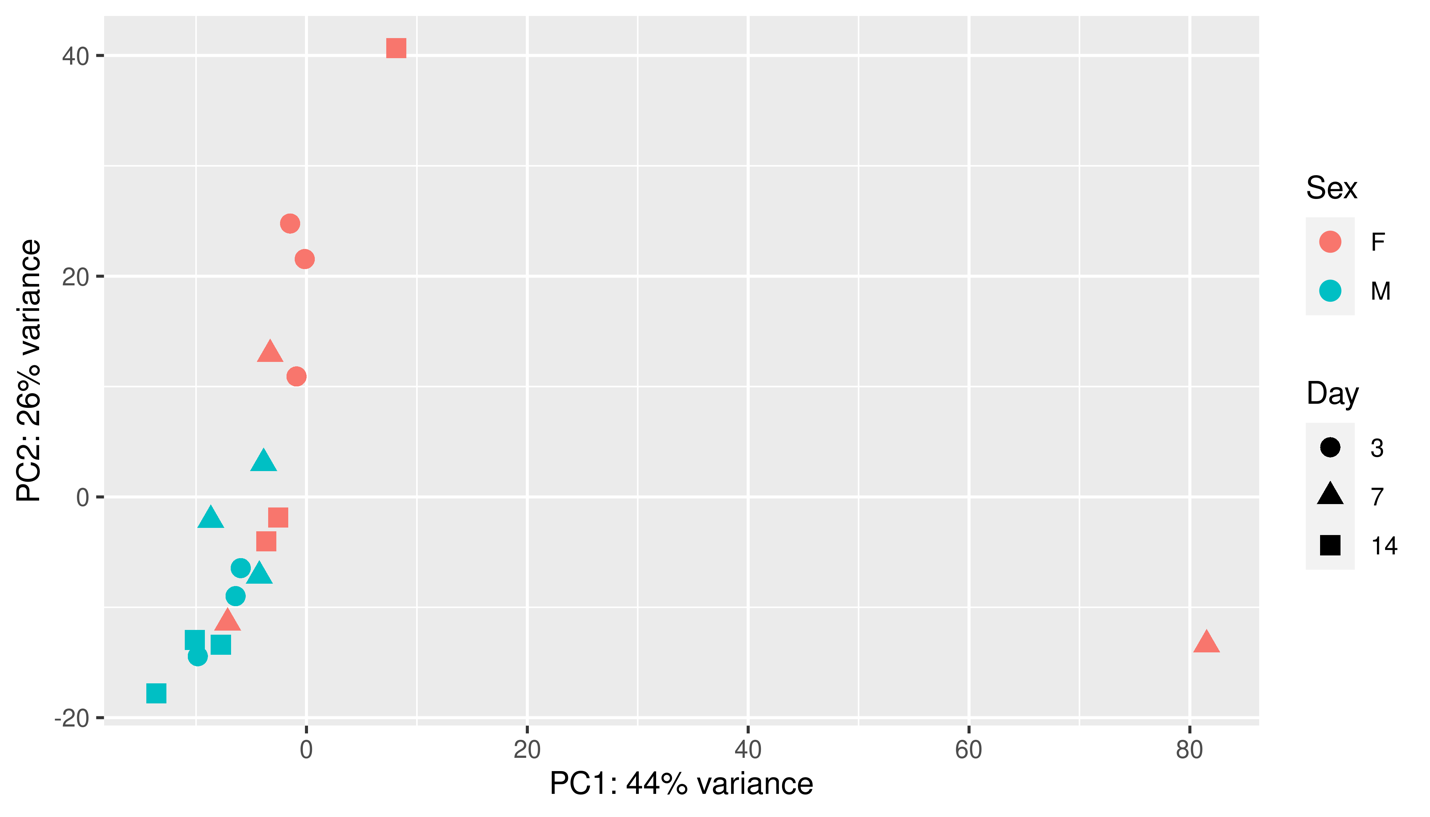


**Fig. S4** Principal component analysis of gene expression profiles for each sample by sex and age before outliers are removed (N = 18). One replicate within the 7-day female condition (rightmost point) and one replicate of the 14-day female condition (topmost point) were considered outliers and removed from downstream analyses. Colors represent sexes, and shapes represent age groups


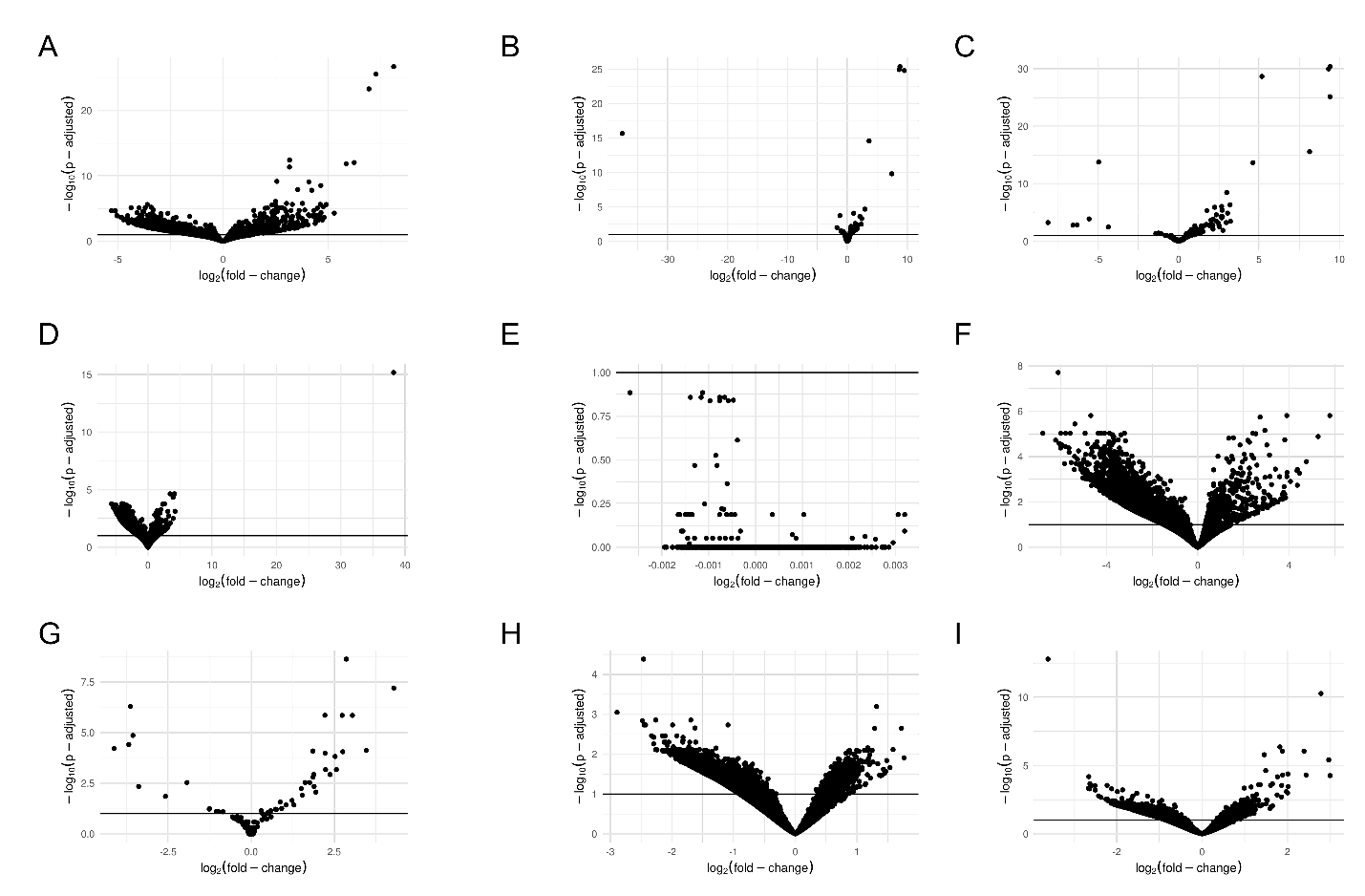


**Fig. S5** Volcano plots with FDR control comparing sex and days of exposure: (a) 3-day females vs. 3-day males, (b) 7-day females vs. 7-day males, (c) 14-day females vs. 14-day males, (d) 3-day vs. 7-day females, (e) 7-day vs. 14-day females, (f) 3-day vs. 14-day females, (g) 3-day vs. 7-day males, (h) 7-day vs. 14-day males, and (i) 3-day vs. 14-day males. Each scatter point represents a gene that 1) passed the low count filter and 2) was not detected as an outlier by DESeq2 in R. Horizontal black lines represent the significance threshold (p = 0.1). There are (a) 1948, (b) 82, (c) 87, (d) 1767, (e) 0, (f) 3148, (g) 47, (h) 3487, and (i) 3321 genes above the significance threshold


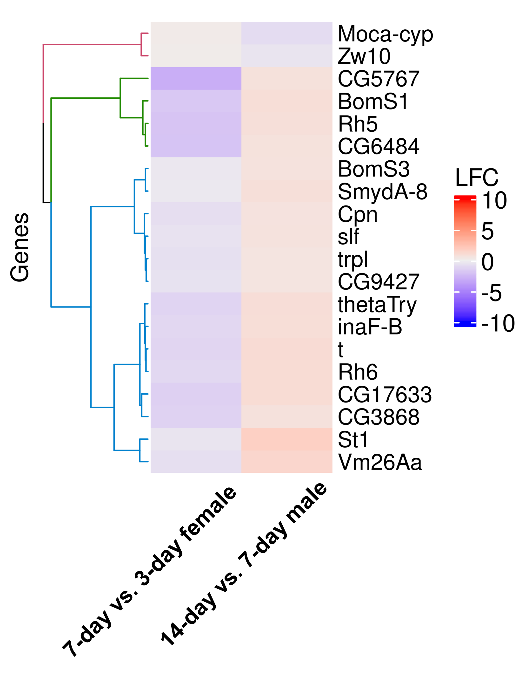


**Fig. S6** Of the 1890 DEGs identified in F3v7 and M7v14 comparisons, 20 (1.1%) exhibit contrasting gene regulation up- and down-regulation between the older and the younger flies. Heatmap and hierarchical clustering show genes that exhibit contrasting up- and down-regulation. Log2(fold-change) (LFC) is the later *vs*. the earlier time point, so genes with positive LFC increased in expression over time. Red cell colors depict upregulation over time, and blue colors depict downregulation over time


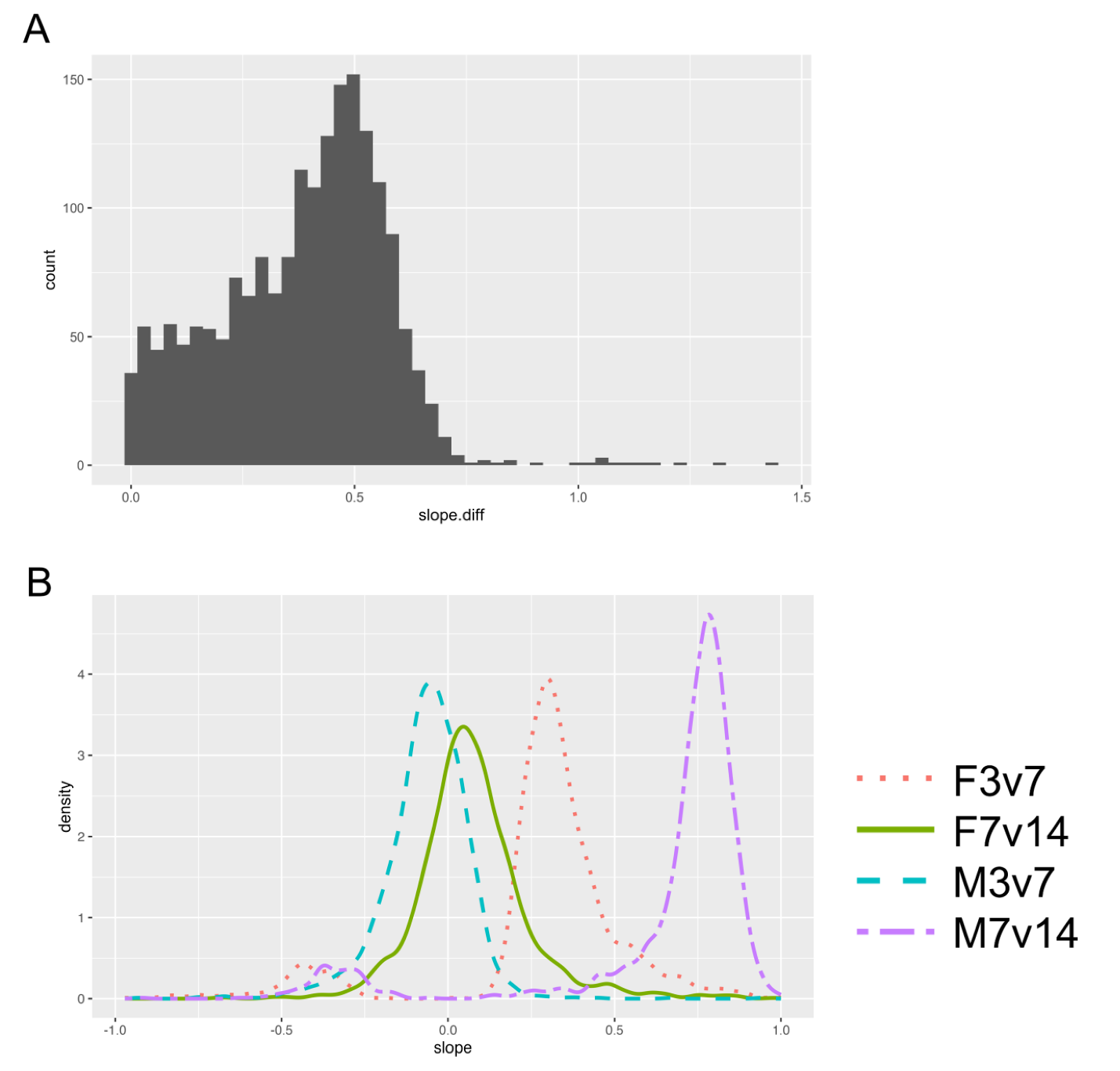


**Fig. S7** (A) Differences between 3-to-7-day and 7-to-14-day fruit flies’ gene expression slopes and (B) slopes by sex and time range, with normalization to a maximum of one before further correction. The slopes are uncorrected and do not account for the time range differences between 3-to-7 days and 7-to-14 days. Colors indicate sex and time range​


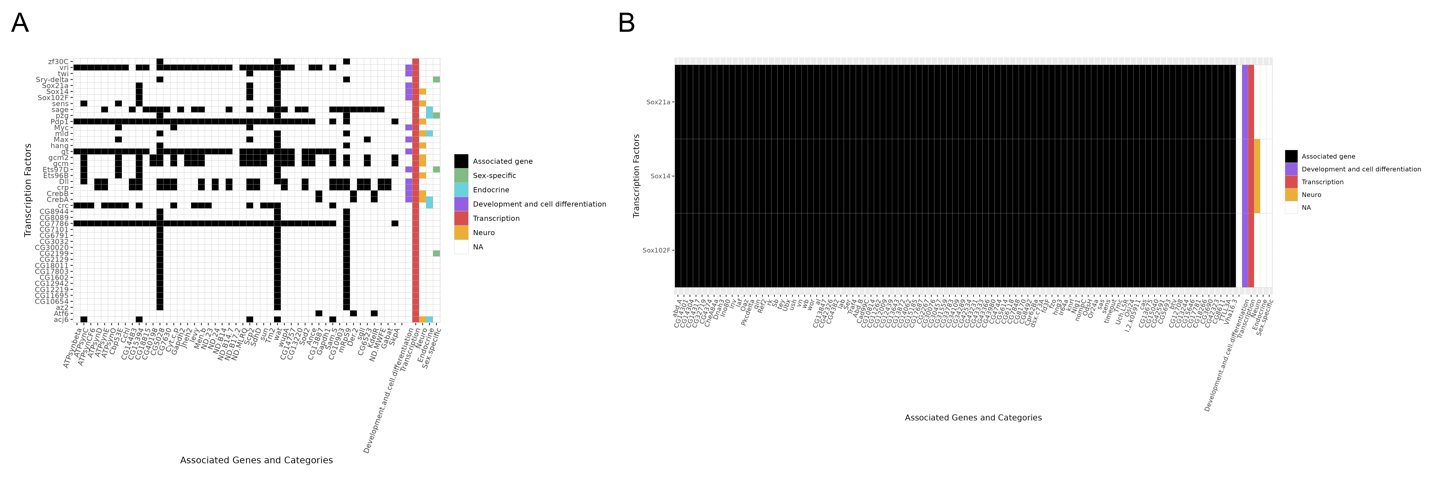


**Fig. S8** Categorization of high-confidence transcription factors: Cell plots of transcription factors enriched among delayed DEGs and their associated genes. (a) Delayed genes whose expression decreased over time, and (b) delayed genes whose expression increased over time. Transcription factors were categorized manually by function: sex-specific, endocrine, development and cell differentiation, transcription, and neuro. A transcription factor was considered enriched if it matched to an over-represented motif with high confidence


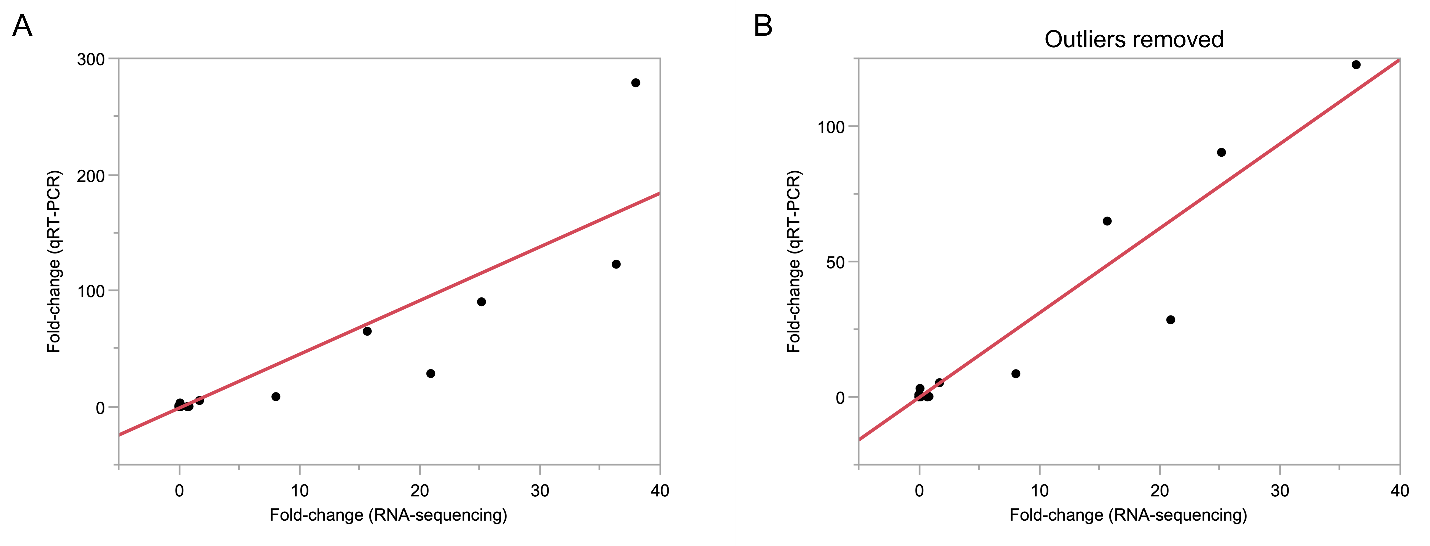


**Fig. S9** Correlation between RNA-sequencing and qRT-PCR for 3, 7, and 14-day female and male *Drosophila melanogaster,* with (a) all genes included and (b) *Jon65Aiv* excluded. RNA-sequencing data was normalized by TMM. Both RNA-sequencing and qRT-PCR were normalized to the housekeeping gene: *RpL32*. The following ten genes were compared for each condition: *SA-2*, *solo*, CG10182, *Yp1*, *Yp3*, *Acp70A*, CG43055, *Jon65Aiv*, CG5107, and CG13428. (a) Including all genes: R-squared = 0.810, R-squared-adjusted = 0.807, Pearson’s correlation = 0.90, p < 0.0001. (b) Excluding *Jon65Aiv*: R-squared = 0.919, R-squared-adjusted = 0.918, Pearson’s correlation = 0.959, p < 0.0001
